# Registration Difficulties—V

**Published:** 1908-12-05

**Authors:** 


### Nursing Administration.

### REGISTRATION DIFFICULTIES—V.

The size of a training school is clearly not the most important factor to be considered in gauging its excellence. Some of the worst training to which it is possible under modern conditions a nurse can be subjected has been carried on in establishments of an imposing description. When for any cause things go wrong in a large training school, and people begin to do what is right in their own eyes, there is hardly any limit to the extent to which the evil may grow. A probationer may be kept in one department for a year or more, doing routine work which has but slender relation to nursing, and may leave at the end of her term only half instructed in the most elementary nursing duties. Others may acquire a slap-dash manner, never lost through life, of doing things which call for extreme accuracy. Others slide unreproved into exactly the wrong manner towards their superiors, towards the medical men, and towards the patients. The wards get to have a neglected look, the patients become discontented and worried, and even the nurses' caps are not set straight on their heads. And yet the training school, in the eyes of the public, may carry sufficient weight to admit those trained under such auspices to the most coveted services, and will serve as a passport to the nurse through life. These facts are not sufficiently considered by those who maintain that our present system of training nurses under a kind of private competition between one institution and another is in no need of revision. But the desired end of safeguarding the nurse's training at every point cannot be attained by setting up a standard in which the size of the institution holds the front place among the conditions laid down as essential for the training school.

The one essential to success in a small hospital which is to give satisfactory training to probationers is a matron trained herself in a good training school with a high standard of work. This appears to be very little understood by those responsible for the management of small hospitals, for the salaries offered as a rule for these posts are absolutely inadequate, and the bad repute of many cottage hospitals in regard to nursing matters makes it evident that the right people are not always secured. With a good nurse at the head of the hospital, and the beds, however small their number, kept pretty constantly full, there are the materials for training a probationer. There is hardly any limit to the knowledge of nursing which a skilled nurse can impart under such conditions. In a ward of five beds, three of which are filled, every quality which a nurse ought to possess in private work can be developed. With two such wards, for male and female patients respectively, the foundations of a private nurse's education can be laid with a security and precision which cannot be outdone in any big institution. It is to be believed that were such training taken into account by any responsible authority it would speedily assert its claim to consideration. It is not complete, and it is never likely to be reckoned as complete. But we maintain that it is, or ought to be a good beginning, and that instead of being thrust aside as a disadvantage, if anything, to the budding nurse, it ought to be regularised and brought into line with training offered in other institutions. The fact that it is not complete has little to do with the want of opportunity for seeing a great variety of medical and surgical cases. Too much is made of the possible gaps in the experience of nurses trained in small hospitals; they are as nothing to the gaps in the experience of nurses who are trained in institutions in which the staff is so large that all cannot be afforded suitable opportunities. The want of completeness in such an accurate, if limited, range of instruction consists in the environment rather than in the number of things learnt and practised. It is not possible, or desirable, to keep probationers more than two years in a very small institution. Unless exceptionally stupid, they must at the end of that time have learned all that the matron has to teach them at the bedside of the patient, and the management of a ward, as well as some of the more complex duties of the matron. They will have learned how to act as housekeeper, how to manage the linen, how to write letters, make out receipts, and receive visitors. They will not be quite on the usual pattern, for the individual training they have received will tend to develop personality. But so far as they go they will be sure of their knowledge, and it will be of a varied character. Finally, they will have imbibed from their chief a burning desire for further experience. To insist that the nursing qualifications of women who have been through such a careful course as has been indicated are nought, that their time has been thrown away from a nursing point of view, and that they must start again on a three years' course as absolute novices or be branded as impostors, is as futile as it is unjust.

It is essential, in our opinion, to any equitable system of State registration for nurses that the rights of the small hospitals should be safeguarded. The only redeeming point in a qualifying examination would be to give probationers trained under less advantageous conditions than those afforded in the large training schools, an opportunity of proving their worth. If this is to be cut away by hard and fast regulations determining an imaginary line, beyond which neither the skill of pupil or teacher is to prove of any avail, a serious blow will be struck at the efficiency of countless establishments now doing good work. The problem of dealing fairly with the smaller institutions, and with the special hospitals, while yet not establishing regulations which shall tend to lower the general standard of training throughout the country, is by far the most acute which would await the members of the prospective Council of Registration. We believe it to be a problem with which a State constituted authority is not well adapted to grapple.

